# Classification and adaptive behavior prediction of children with autism spectrum disorder based upon multivariate data analysis of markers of oxidative stress and DNA methylation

**DOI:** 10.1371/journal.pcbi.1005385

**Published:** 2017-03-16

**Authors:** Daniel P. Howsmon, Uwe Kruger, Stepan Melnyk, S. Jill James, Juergen Hahn

**Affiliations:** 1 Department of Chemical and Biological Engineering, Rensselaer Polytechnic Institute, Troy, New York, United States of America; 2 Center for Biotechnology and Interdisciplinary Studies, Rensselaer Polytechnic Institute, Troy, New York, United States of America; 3 Department of Biomedical Engineering, Rensselaer Polytechnic Institute, Troy, New York, United States of America; 4 Department of Pediatrics, University of Arkansas for Medical Sciences, Little Rock, Arkansas, United States of America; Centre for Research and Technology-Hellas, GREECE

## Abstract

The number of diagnosed cases of Autism Spectrum Disorders (ASD) has increased dramatically over the last four decades; however, there is still considerable debate regarding the underlying pathophysiology of ASD. This lack of biological knowledge restricts diagnoses to be made based on behavioral observations and psychometric tools. However, physiological measurements should support these behavioral diagnoses in the future in order to enable earlier and more accurate diagnoses. Stepping towards this goal of incorporating biochemical data into ASD diagnosis, this paper analyzes measurements of metabolite concentrations of the folate-dependent one-carbon metabolism and transulfuration pathways taken from blood samples of 83 participants with ASD and 76 age-matched neurotypical peers. Fisher Discriminant Analysis enables multivariate classification of the participants as on the spectrum or neurotypical which results in 96.1% of all neurotypical participants being correctly identified as such while still correctly identifying 97.6% of the ASD cohort. Furthermore, kernel partial least squares is used to predict adaptive behavior, as measured by the Vineland Adaptive Behavior Composite score, where measurement of five metabolites of the pathways was sufficient to predict the Vineland score with an *R*^2^ of 0.45 after cross-validation. This level of accuracy for classification as well as severity prediction far exceeds any other approach in this field and is a strong indicator that the metabolites under consideration are strongly correlated with an ASD diagnosis but also that the statistical analysis used here offers tremendous potential for extracting important information from complex biochemical data sets.

## Author summary

Autism spectrum disorder (ASD) encompasses a family of neurological disorders characterized by limited social interaction and restricted repetitive behaviors. The number of children diagnosed with ASD has grown exponentially over the last four decades and is now estimated to affect ∼1.5% of children. Although ASD is currently diagnosed and treated based solely on psychometric tools, a biochemical view applicable to at least a subset of ASD cases is emerging. Abnormalities in folate-dependent one carbon metabolism and transsulfuration pathways can summarize a large number of observations of genetic and environmental effects that increase ASD predisposition. However, these complex, highly interconnected pathways require more advanced statistical models than the typical univariate models presented in the literature. Therefore, we developed multivariate statistical models that classify participants based on their neurological status and predict adaptive behavior in ASD. We emphasize that these models are cross-validated, helping to ensure that the results will generalize to new samples. The models developed herein have much stronger predictability than any existing approaches from the scientific literature.

## Introduction

Autism Spectrum Disorder (ASD) encompasses a large group of early-onset neurological diseases characterized by difficulties with social communication/interaction and expression of restricted repetitive behaviors and interests [1]. In addition to these defining behavioral symptoms, individuals with ASD frequently have one or more co-occurring conditions, including intellectual disability, ADHD, speech and language delays, psychiatric diagnoses, epilepsy, sleep disorders, and gastrointestinal problems [2–5]. ASD affects ∼1.5% of the population and affects males disproportionately [6–8]. It is associated with an impaired quality of life [9] and the lifetime cost of supporting an individual with ASD amounts to $1.4–2.4MM, depending on co-existing disorders [10].

It is generally acknowledged that ASD has a strong genetic component, but environmental effects have also recently emerged as important contributors to the etiology and pathophysiology of ASD in at least a subpopulation of cases. Early twin studies suggested that the heritability of ASD was 80–90% [11]; however, twin studies since 2010 suggest a lower heritability of only 37–55% [12, 13]. Despite this high genetic association, only 15% of ASD cases have a known genetic source [1]. Although genetic studies continue to provide new evidence for contributing factors to ASD etiology [14], environmental effects such as maternal/paternal age, toxic chemical exposure, maternal rubella infection, etc. are also emerging as key factors contributing to ASD liability [13].

**Fig 1:**
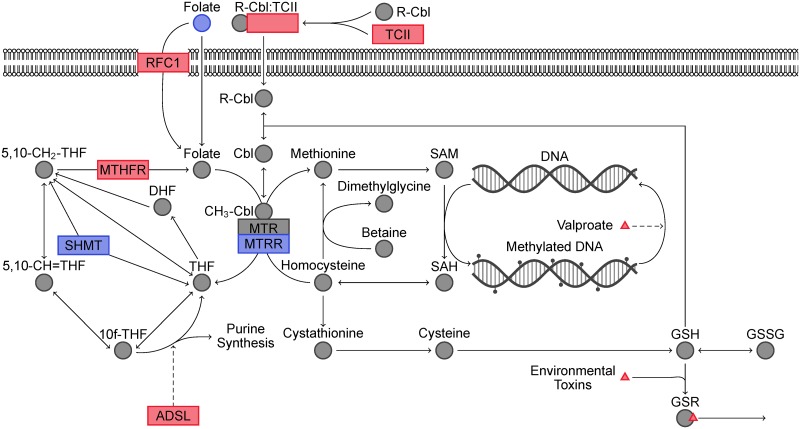
Illustration of folate-dependent one-carbon metabolism and transsulfuration pathways. Genetic and environmental effects that increase ASD predisposition are shown in red whereas those that decrease ASD liability are shown in blue.

No generally accepted biomarkers for the diagnosis or diagnosis of the severity of ASD exist to date. Instead, diagnostic evaluation involves a multi-disciplinary team of doctors usually including a pediatrician, psychologist, speech and language pathologist, and occupational therapist. Despite this current state of the art, work in identifying biomarkers that can support the diagnosis process is ongoing. In particular, abnormalities in folate-dependent one-carbon metabolism (FOCM) and transsulfuration (TS) likely contribute to the genetic and environmental predisposition to ASD [15]. FOCM contributes to epigenetic gene expression through DNA methylation and TS is the major contributor to intracellular redox status. An illustration of these pathways overlaid with genetic and environmental contributions to ASD predisposition is presented in Fig 1.

Mutations or altered expression levels of several genes in these pathways have been associated with increased risk of ASD. Adenylosuccinate lyase (ADSL) deficiency leads to a purely genetic form of autism by re-directing a large proportion of FOCM toward purine synthesis to compensate for a reduction in *de novo* purine synthesis [15, 16]. Methylenetetrahydrofolate reductase (MTHFR) is responsible for generating 5-methyltetrahydrofolate, which in turn is responsible for re-methylating homocysteine to methionine. In particular, the C677T polymorphism has been shown to increase ASD liability, especially in countries where prenatal folate supplementation is low [17]. Limited evidence linking mutations in reduced folate carrier (RFC1) [18, 19], transcobalamin II (TCII) [18], serine hydroxymethyltransferase I (SHMT1) [20], 5-methyltetrahydrofolate-homocysteine methyltransferase reductase (MTRR) [18, 20], and catechol-O-methyltransferase (COMT) [18, 21] to altered prevalence of ASD has also been presented, although these contributions to ASD liability are currently contested [22].

Evidence for the association between environmentally-rooted FOCM/TS dysfunction and ASD predisposition can be seen in prenatal valproate and toxic chemical exposure as well as lack of maternal folate supplementation. Maternal valproate use during pregnancy has been associated with higher incidence rates of ASD [23, 24] and *in utero* valproate exposure has been used to develop rodent models of autism [25]. Valproate exposure causes DNA hypomethylation [26, 27] in key neurodevelopmental processes that have been mitigated by folate supplementation [28]*in vitro*. Other chemicals such as heavy metals, ethyl alcohol, pesticides, phthalates, polychlorinated biphenyls, and traffic-related air pollution (TRAP) have also been shown to affect neurodevelopment and increase ASD liability [13, 29]. These organic toxins induce oxidative stress and heavy metals disrupt transsulfuration by binding glutathione, the major contributor to intracellular redox homeostasis [30]. Additionally, glutathione is an important regulator in the intracellular processing of methylcobalamin (vitamin B_12_), an essential cofactor for methionine synthase and the TS pathway [31]. Air dispersion models coupled with traffic patterns/roadway geometry, meteorological data, and vehicle emission data have been used to find a dose response between ASD prevalence and TRAP exposure [32]. Additionally, common organic pollutants have been associated with increased autism severity in children on the autism spectrum [33]. Two independent studies linked maternal folate supplementation to a reduced risk of having a child with ASD [34, 35]. This protective effect is usually attributed to the involvement of FOCM in early epigenetic regulation of neurodevelopment and neural tube formation [21, 36]. For a more complete description of the evidence for the potential contributions of FOCM/TS dysfunction to the ASD phenotype, see the excellent review by Deth *et al*. [15].

Although differences between FOCM and TS pathways in children with ASD versus neurotypical controls have been shown previously [18, 37, 38], investigators have struggled with identifying a single, predictive measurement of these pathways that separates individuals with ASD from neurotypical controls or that correlates well with ASD severity. However, in many complex problems one particular measurement may be insufficient and important information can only be extracted by using multivariate statistical analysis. Indeed, incorporating multiple measurements of environmental toxins has been shown to increase the separability of control and ASD participants [39] and better predict autism severity [33, 39].

Latent variable techniques enable the discovery of important multivariate interactions, leading to improved classification and regression performance. Furthermore, latent variable techniques allow assessing the importance of individual variables and are more robust to uninformative variables. One popular latent variable technique for classification problems is Fisher Discriminant Analysis (FDA), which achieves an optimal linear separability using a typically small set of latent variables that are linear combinations of the original variable set. FDA has a long history in biological classification problems and was first used by Rao in 1948 to interpret anthropological data [40]. Extensions of FDA, such as Kernel FDA (KFDA), exist which can take nonlinear relationships into account for classification [41]. Latent variable regression techniques include partial least squares (PLS) and its nonlinear counterpart kernel PLS (KPLS) [42, 43]. Using FDA for classification and KPLS for regression allow multivariate interactions to surface, which are often hidden when only univariate analysis is considered. To guarantee a statistically independent assessment of the multivariate classification and regression models, the presented study utilizes a cross-validatory approach, where the set of samples used for model identification does not contain samples to evaluate the performance of the identified models.

The presented work makes use of these advanced modeling and statistical analysis tools to examine metabolite data of the FOCM/TS pathway in neurotypical participants (NEU) and those on the autism spectrum (ASD) as well as their siblings (SIB). Using FDA, it is possible to clearly distinguish the participants on the spectrum from their neurotypical peers and KPLS unveils a strong correlation between metabolite concentrations of these pathways and adaptive behavior as measured by the Vineland Adaptive Behavior Composite. This work not only analyzes the largest data set of its kind of these pathways in the scientific literature [38], but also results in the strongest evidence to date of the association of FOCM/TS dysfunction with ASD.

## Results

### Classification into ASD, NEU, and SIB cohorts

**Fig 2:**
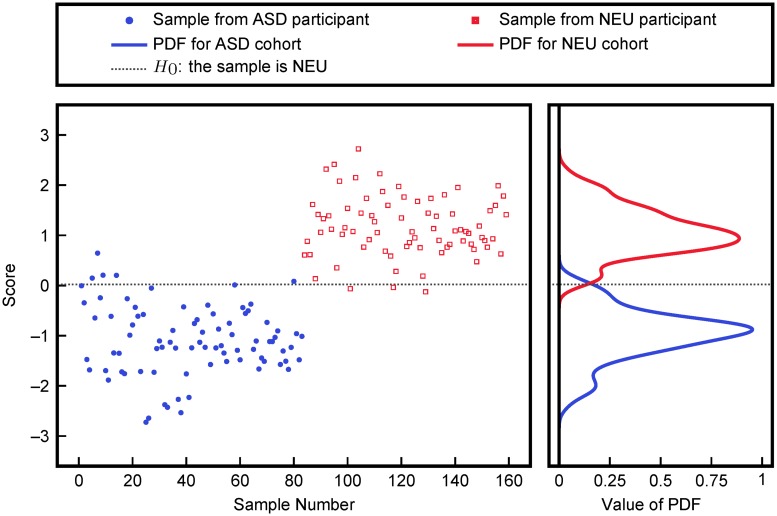
Classification into ASD and NEU cohorts using FDA on all FOCM/TS metabolites. The plotted scores were obtained via cross-validation and the probability distribution functions were obtained from fitting.

Associating dysfunction of FOCM/TS pathways with ASD requires a distinction between or separation of ASD and NEU groups based on FOCM/TS metabolites. Therefore, cross-validatory FDA was performed using measurements of the FOCM/TS metabolites listed in Table 1. A linear classifier based on these FDA scores is then used to classify ASD and NEU participants. FDA scores and estimated probability distribution functions (PDFs) are provided in Fig 2. The cross-validated misclassification rates of only 4.9% and 3.4% for the NEU and ASD samples, respectively, eliminated more complex, nonlinear KFDA analysis from consideration.

**Table 1:** FOCM/TS metabolites considered for analysis.

Methionine	SAM	SAH
SAM/SAH	% DNA methylation	8-OHG
Adenosine	Homocysteine	Cysteine
Glu.-Cys.	Cys.-Gly.	tGSH
fGSH	GSSG	fGSH/GSSG
tGSH/GSSG	Chlorotyrosine	Nitrotyrosine
Tyrosine	Tryptophane	fCystine
fCysteine	fCystine/fCysteine	% oxidized glutathione

**Fig 3:**
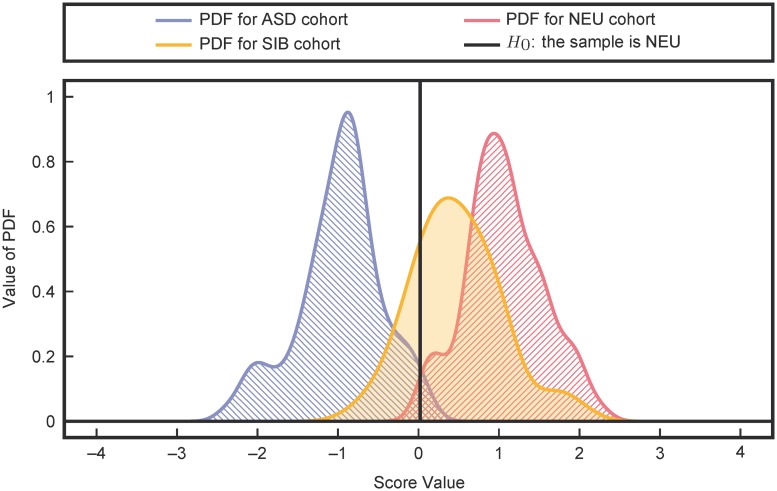
Classification performance on the SIB cohort (yellow). There is significantly more overlap of the SIB cohort with the NEU cohort (red) than with the ASD cohort (blue).

The performance of the classifier was then evaluated on the SIB cohort, a more challenging classification problem due to partially shared genetic and environmental effects with the ASD cohort. Using all measurements in Table 1, an FDA model was trained to separate the ASD and NEU cohorts. Then, the trained FDA model was used to evaluate the SIB cohort (which was not used for training). The resulting separation of ASD, NEU, and SIB presented in Fig 3 shows a slight increase in the overlap with the ASD cohort when compared with the performance of the ASD vs. NEU classification. Furthermore, the SIB PDF shows significantly more overlap with the NEU PDF than the ASD PDF. These results support the hypothesis proposed by James *et al* [38] that the siblings of the participants on the spectrum have FOCM/TS metabolite profiles that are significantly more similar to their neurotypical peers than their siblings, even though genetically they are likely closer to their siblings than participants in the neurotypical control group.

### Analysis of important metabolites for classification

**Fig 4:**
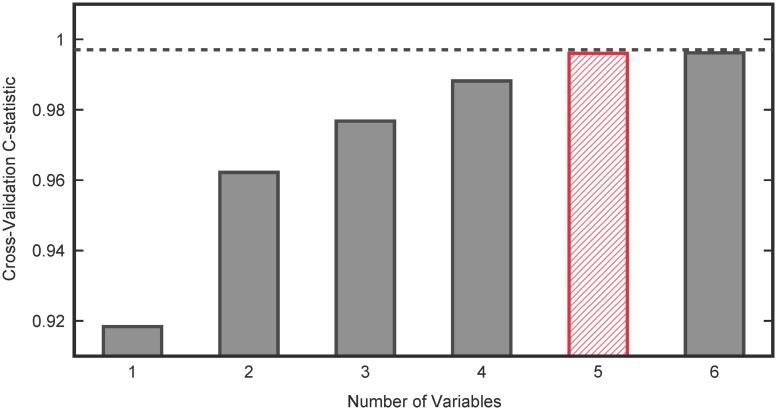
Selecting the Number of Variables for FDA based on C-statistic. Five variables were found to be sufficient for separating the ASD and NEU groups while an additional two variables (totaling seven variables) were incorporated to retain separation between ASD and SIB cohorts.

The simultaneous use of multiple measurements promises to increase the separability of the cohorts; however, increasing the number of measurements increases the number of parameters in the projection vector *w* that maximizes the separability of the two groups (see Materials and methods). Although cross-validation can help mitigate these effects, the increased number of parameters can lead to over-fitting, which would indicate good performance for separation on the existing data set, but poor separation performance when the analysis results are translated to new data. These over-fitting problems can be further mitigated by selecting only the minimum number of variables required to adequately separate the two groups. Therefore, all combinations of up to six variables were evaluated for separability. Select combinations of higher numbers of variables were chosen in a greedy fashion to sequentially add measurements that best improve the separation of the best six variables. Cross-validatory FDA was performed on all variable combinations and probability distribution functions (PDFs) of the FDA scores of the two cohorts were estimated. A receiver-operating-characteristic (ROC) curve was generated based on these PDFs. The C-statistic of the ROC curve provides a measure of the ability of the classifier to separate into ASD and neurotypical groups. A C-statistic of 0.5 represents random classification and a C-statistic of 1.0 represents perfect classification. Fig 4 plots the maximum C-statistic for all combinations of a given number of variables. As the number of variables increases, the C-statistic increases, saturates at 0.997, and then slightly decreases when over-fitting occurs.

**Fig 5:**
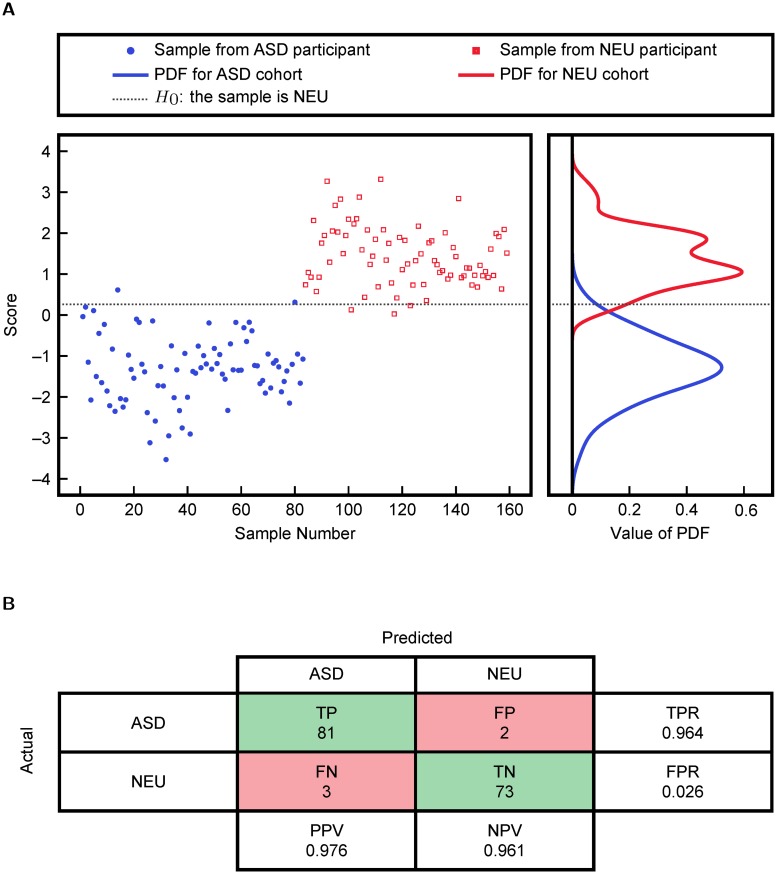
FDA analysis and binary classification using the variables DNA methylation, 8-OHG, Glu.-Cys., fCystine/fCysteine, % oxidized glutathione, Chlorotyrosine, and tGSH/GSSG. (a) individual cross-validated FDA scores and fitted probability distribution functions and (b) the cross-validated confusion matrix for separation of ASD and neurotypical (NEU) groups. TPR = TP/(TP + FN) is the True Positive Rate, FPR = FP/(FP + TN) is the False Positive Rate, PPV = TP/(TP + FP) is the Positive Predictive Value, and NPV = TN/(TN + FN) is the Negative Predictive Value.

From these results, five variables (DNA methylation, 8-OHG, Glu.-Cys., fCystine/fCysteine, % oxidized glutathione) were considered for further analysis; however, it should be noted that select variable combinations distinct from this one provided similar performance for separating ASD and NEU participants. Chlorotyrosine and tGSH/GSSG were added to this set to improve separability of the ASD and SIB groups, increasing the number of metabolites under consideration to seven. The separability of the final minimal classifier based on these seven variables is presented in Fig 5 with Type I and Type II error plots in S1 Fig.

### Prediction of adaptive behavior in ASD

**Fig 6:**
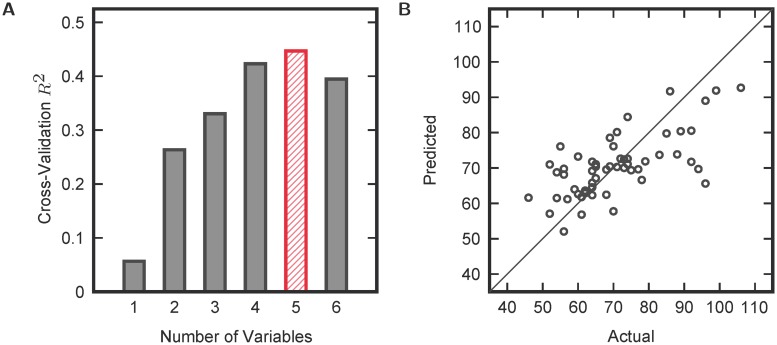
KPLS regression results. (a) maximum cross-validated *R*^2^ for a given number of variables and (b) cross-validated model predictions versus actual data points for the best combination of five variables (GSSG, tGSH/GSSG, Nitrotyrosine, Tyrosine, and fCysteine).

In addition to separation into neurologically distinct cohorts, metabolites in the FOCM/TS pathway were investigated for predictability of adaptive behavior. Due to the inter-dependency of pathway metabolites and possible nonlinear effects on psychological outcomes, nonlinear regression via KPLS was used to evaluate the ability of pathway metabolites to predict adaptive behavior in ASD (as measured by the Vineland Adaptive Behavior Composite score). Just as was done in the FDA analysis, all combinations of a given number of variables were evaluated for predictability. The cross-validatory *R*^2^ of the regression was then used to determine the optimal number of variables in the regression analysis. From the results in Fig 6, the *R*^2^ begins to decrease when more than five variables are used in the KPLS analysis. The maximum cross-validatory *R*^2^ was 0.45, corresponding to the KPLS model with the variable combination GSSG, tGSH/GSSG, Nitrotyrosine, Tyrosine, and fCysteine used as inputs. These regression results are plotted in Fig 6. (It is important to note that a few other variable combinations provided similar results, but only the best regression model is illustrated for clarity.) This strong correlation even after cross-validation indicates the importance of FOCM/TS dysfunction in the pathophysiology of ASD.

## Discussion

The multivariate statistical analysis presented herein provides unprecedented quantitative classification results for separating participants into ASD and NEU cohorts based solely on biochemical data. Existing analyses report differences in mean metabolite levels or provide qualitative illustrations of separating these two groups based on FOCM/TS metabolites [18, 37, 38]. However, these strategies are not designed for classification and thus fail to successfully classify participants. Here, FDA on seven metabolites allows sufficient separation such that a linear classifier can correctly resolve 96.9% of participants. Such low misclassification rates dissuaded the use of more complex, nonlinear methods such as KFDA. Although FOCM/TS dysfunction likely does not completely detail ASD etiology, this biochemical analysis approaches the accuracy needed for a clinical diagnostic tool.

Classification performance on the SIB group fortifies the argument for FOCM/TS involvement in ASD since the large degree of shared genetic and environmental effects with the ASD population only slightly worsens the separation. The sibling recurrence rate for ASD is estimated to be 6.9–18.7% [7, 44, 45] and many siblings perform behaviorally and/or cognitively at intermediate levels between those of ASD and NEU cohorts [45–47] or express traits characteristic of ASD [47–49]. Therefore, the classification performance placing the SIB group between the ASD and NEU groups, albeit much closer to the NEU group, is consistent with the broader scientific literature on psychometric analysis of siblings of people with ASD. Future work would benefit from assessing the SIB and NEU groups on measurements of the Broader Autism Phenotype to validate these hypotheses on mild FOCM/TS dysfunction in the SIB group.

Comparison or meta-analysis of regression analyses across studies is difficult due to differences in metabolites measured, origin of metabolites, available psychometric data, and metrics of model performance. It is emphasized that extreme caution should be used when evaluating fitted versus cross-validated metrics; for example, in [39], the best linear model can achieve a fitting *R*^2^ of 0.296, while obtaining a cross-validated *R*^2^ of only 0.192. In general, fitting results always surpass cross-validation results; nevertheless, the top-performing KPLS model in this study achieved a cross-validatory *R*^2^ of 0.45 due to its ability to reflect nonlinear behaviors/interactions, which surpasses or compares with previous fitting [50, 51] and cross-validated results [39].

Nonlinear regression analysis of FOCM/TS metabolites enables prediction of key FOCM/TS metabolites that are associated with adaptive behavior in ASD. Based upon all variable combinations evaluated in the KPLS regression analysis, top-performing models always incorporated (1) nitrotyrosine, (2) tyrosine, (3) fGSH or tGSH/GSSG, and (4) fCysteine or fCystine/fCysteine. Interestingly, these variables are affected by high quality vitamin supplementation that also decreases ASD severity in at least a subset of cases [51–53]. While this forms an intriguing direction for future studies, it should be noted that these studies should be replicated and empirically tested on a wider scale before more definite conclusions can be drawn. Furthermore, this approach can be extended to include other psychometric instruments (e.g. the Autism Diagnostic Observation Schedule (ADOS) or Childhood Autism Rating Scales (CARS)) that are more appropriate for diagnosis of ASD.

Developmental pediatricians, psychologists and other professionals can effectively use the wealth of information provided by psychometric instruments to diagnose and evaluate patients with ASD. However, these tests can rarely diagnose children under two years old since they are based solely on behavioral assessment. As it is generally acknowledged that an earlier diagnosis can lead to a more favorable outcome in the long run [54], the identification of biomarkers which can be used in conjunction with psychometric measurements would be of significant importance for ASD diagnosis. Furthermore, identification of these biomarkers can facilitate the understanding of these complex disorders, which offers significant potential for developing intervention strategies targeted to normalize these biomarkers in the future. However, it is important to note that these biomarkers may not simply be measurements of certain metabolites but may require nonlinear statistical analysis of the measurements, as is done in this work.

## Materials and methods

### Description of data

The data used in this study comes from the Arkansas Children’s Hospital Research Institute’s autism IMAGE study [38]. The protocol was approved by the Institutional Review Board at the University of Arkansas for Medical Sciences and all parents signed informed consent. The interested reader is referred to [38] for detailed study design, including demographic information and inclusion/exclusion criteria. Briefly, children between the ages of 3 and 10 years were enrolled to assess levels of oxidative stress. ASD was defined by the *Diagnostic and Statistical Manual for Mental Disorders, Fourth Edition*, the Autism Diagnostic Observation Schedule (ADOS), and/or the Childhood Autism Rating Scales (CARS; score > 30). FOCM/TS metabolites from 83, 47, and 76 case (ASD), sibling (SIB), and age-matched control (NEU) children, respectively, were used for classification. The metabolites under investigation are tabulated in Table 1 and additional details of these measurements and derivations are presented in [38]. Of the 83 participants on the autism spectrum, 55 also had Vineland II Scores recorded for use in regression analysis (range 46–106). The Vineland Adaptive Behavior Composite evaluates adaptive skills across the domains of communication, socialization, daily living skills, and motor skills through a semi-structured caregiver interview [55]. Data are available in S1 Dataset.

### Fisher Discriminant Analysis

Fisher Discriminant Analysis (FDA) is a dimensionality reduction tool that seeks to maximize differences between multiple classes. Specifically, for *n* samples of *m* measurements associated with *k* different classes, the between cluster variability *S*_*B*_ is defined to be

$$\begin{array}{c} S_{B}=\sum_{i=1}^{k}n_{i}\left({\bar{x}}_{i}-\bar{x}\right){\left({\bar{x}}_{i}-\bar{x}\right)}^{T} \end{array}$$

where ${\bar{x}}_{i}$ represents the mean vector of class *i*, $\bar{x}$ represents the mean vector of all samples, and *n*_*i*_ represents the number of samples in class *i*. The within cluster variation is defined as

$$\begin{array}{c} S_{W}=\sum_{i=1}^{k}n_{i}\sum_{j\in i}\left(x_{j}-{\bar{x}}_{i}\right){\left(x_{j}-{\bar{x}}_{i}\right)}^{T} \end{array}$$

where *x*_*j*_ represents an individual sample. FDA seeks to find at most *k* − 1 vectors that maximize

$$\begin{array}{c} J\left(w\right)=\frac{w^{T}S_{B}w}{w^{T}S_{W}w} \end{array}$$

In other words, FDA seeks to find linear combinations of variables that project samples in the same group close to each other and project samples in different groups far away from each other. The solution to this optimization problem is the generalized eigenvectors associated with the *k* − 1 largest generalized eigenvalues of $S_{W}^{-1}S_{B}$.

### Kernel density estimation

Kernel density estimation attempts to determine the underlying probability distribution function from a set of reference samples. The main assumption is that additional samples are likely to be found near the reference samples [56–58]. Using a Gaussian kernel, this assumption is formulated into an algorithm by associating a kernel function

$$\begin{array}{c} K\left(\frac{x-x_{i}}{\sigma }\right) \end{array}$$

with each observation *x*_*i*_. Here, *x* is the additional sample and *σ* is the kernel parameter that controls the shape of the distribution function. The estimated density function $\hat{f}\left(x\right)$ is then given by

$$\begin{array}{c} \hat{f}\left(x\right)=\frac{1}{n\sigma }\sum_{i=1}^{n}K\left(\frac{x-x_{i}}{\sigma }\right) \end{array}$$

where *n* is the number of reference samples. The kernel parameter *σ* is chosen to minimize the mean integrated squared error (MISE) between the unknown density function *f*(*x*) and the estimated density function $\hat{f}\left(x\right)$:

$$\begin{array}{c} \text{MISE}\left(\sigma \right)=\int_{-\infty }^{\infty }{\left(f\left(x\right)-\hat{f}\left(x\right)\right)}^{2} \end{array}$$

using a cross-validatory approach [56].

### Kernel partial least squares

Kernel techniques provide general nonlinear extensions to the popular linear partial least squares (PLS) regression. The KPLS algorithm commences by defining a nonlinear transformation *f* = *ψ*(*x*) on the predictor set *x*. In this work, *ψ*(*x*) is a Gaussian kernel. Rather than regression on *x* as in linear PLS, *y* is regressed onto the high dimensional feature space *f* [42, 43].

### Cross-validation

To avoid over-fitting and over-stating results, leave-one-out cross validation is employed in both the FDA and KPLS analysis. The approach leaves out a single sample, fits an FDA or KPLS model, and evaluates the prediction of the sample left out. This scheme is repeated for each sample.

## Supporting information

S1 Dataset Biochemical and Adaptive Behavior Data from ASD, NEU, and SIB Participants. (CSV)

S1 Fig Type I and II Errors for the Final FDA Model. Cross-validated type I and type II errors for the FDA model using the variables DNA methylation, 8-OHG, Glu.-Cys., fCystine/fCysteine, % oxidized, Chlorotyrosine, and tGSH/GSSG.
(TIF)
