# Supplementary figures and images for: Classification and adaptive behavior prediction of children with autism spectrum disorder based upon multivariate data analysis of markers of oxidative stress and DNA methylation
Source: PLoS Comput Biol. 2017 Mar 16;13(3):e1005385. doi: 10.1371/journal.pcbi.1005385 (PMC5354243; doi:10.1371/journal.pcbi.1005385)

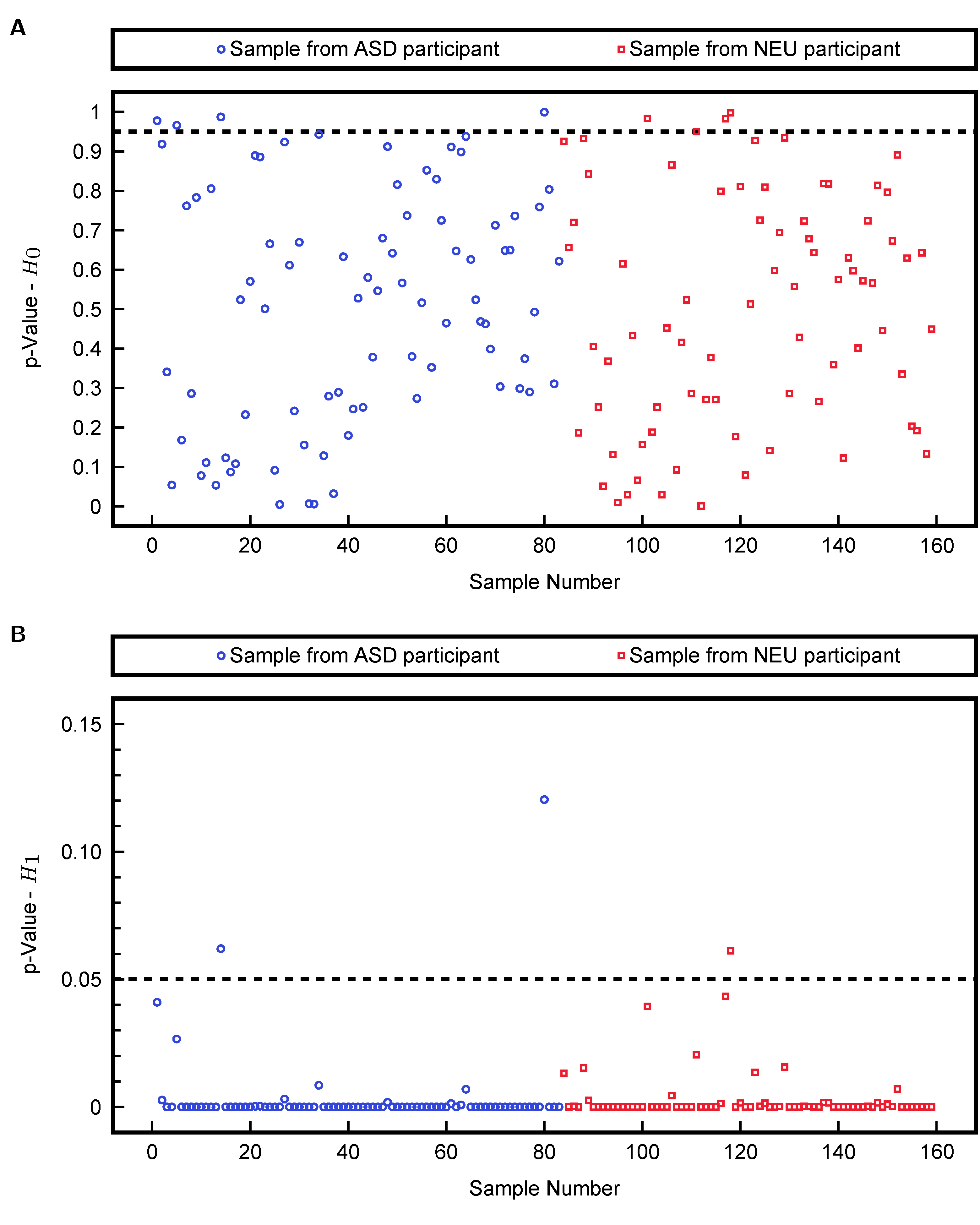

Supplement: S1 Fig — Cross-validated type I and type II errors for the FDA model using the variables DNA methylation, 8-OHG, Glu.-Cys., fCystine/fCysteine, % oxidized, Chlorotyrosine, and tGSH/GSSG. (TIF) [file pcbi.1005385.s002.tif]
